# Anion Exchanger 1b in Stereocilia Is Required for the Functioning of Mechanotransducer Channels in Lateral-Line Hair Cells of Zebrafish

**DOI:** 10.1371/journal.pone.0117041

**Published:** 2015-02-13

**Authors:** Yuan-Hsiang Lin, Giun-Yi Hung, Liang-Chun Wu, Sheng-Wen Chen, Li-Yih Lin, Jiun-Lin Horng

**Affiliations:** 1 Department of Electronic and Computer Engineering, National Taiwan University of Science and Technology, Taipei, Taiwan, ROC; 2 Department of Pediatrics, Taipei Veterans General Hospital, Taipei, Taiwan, ROC; 3 Department of Pediatrics, Faculty of Medicine, National Yang-Ming University, Taipei, Taiwan, ROC; 4 Department of Life Science, National Taiwan Normal University, Taipei, Taiwan, ROC; 5 Department of Anatomy and Cell Biology, School of Medicine, College of Medicine, Taipei Medical University, Taipei, Taiwan, ROC; University of Houston, UNITED STATES

## Abstract

The anion exchanger (AE) plays critical roles in physiological processes including CO_2_ transport and volume regulation in erythrocytes and acid-base regulation in renal tubules. Although expression of the AE in inner-ear hair cells was reported, its specific localization and function are still unclear. Using *in situ* hybridization, we found that the AE1b transcript is expressed in lateral-line hair cells of zebrafish larvae. An immunohistochemical analysis with a zebrafish-specific antibody localized AE1b to stereocilia of hair cells, and the expression was eliminated by morpholino knockdown of AE1b. A non-invasive, scanning ion-selective electrode technique was applied to analyze mechanotransducer (MET) channel-mediated Ca^2+^ influx at stereocilia of hair cells of intact fish. Ca^2+^ influx was effectively suppressed by AE1b morpholino knockdown and inhibitor (DIDS) treatment. Elevating external Ca^2+^ (0.2 to 2 mM) neutralized the inhibition of DIDS. Taken together, this study provides solid evidence to show that AE1b in stereocilia is required for the proper functioning of MET channels.

## Introduction

Anion exchanger 1 (SLC4A1, AE1 or band 3) is a member of the SLC4 bicarbonate transporter family and it electroneutrally exchanges one chloride for one bicarbonate in physiological conditions. AE1 is the main membrane protein in vertebrate erythrocytes, and it carries out several tasks including a respiratory role by improving CO_2_ (HCO_3_^-^) transport and a structural role by linking plasma membranes to the cytoskeleton; it is also involved in volume regulation of erythrocytes [1], [2]. AE1 is also expressed in basolateral membranes of α-intercalated cells in renal tubules and reclaims bicarbonate to the systemic circulation and facilitates acid excretion [3], [4]. Furthermore, AE proteins were found in the mammalian inner ear and were suggested to play a role in maintaining endolymphatic pH [5], [6].

In mammals, hair cells in the inner ear are specialized mechanosensory cells involved in hearing and balance. Hair cells have a special morphological feature of apical hair bundles which consist of stereocilia that contain a mechanotransducer (MET) channel close to their tips and are connected by tip links [7]. Deflection of a hair bundle opens the MET channel and causes Ca^2+^ and K^+^ influx, which activates signal transduction in hair cells [8]. The MET channel is a non-selective cation channel but has particularly high Ca^2+^ permeability. It is also permeable to small organic cations such as FM1–43 and can be blocked by an assortment of agents such as La^3+^, Gd^3+^, amiloride, and aminoglycoside antibiotics [8].

Zebrafish are recognized as a useful *in vivo* model for studying vertebrate hair cells [9], [10], [11]. Unlike mammals whose inner-ear hair cells are embedded in the temporal bone, hair cells of zebrafish are organized into lateral-line neuromasts which are on the embryonic skin and can be easily observed and investigated [12], [13], [14]. Neuromasts contain a core of ~15 hair cells that have a structure and function similar to those of inner-ear hair cells in other vertebrates including humans [9], [10], [11]. For the first time, we recently developed a scanning ion-electrode technique (SIET) to detect MET channel-mediated Ca^2+^ entry at neuromast hair cells of zebrafish. Using a Ca^2+^-selective microelectrode to deflect hair bundles and simultaneously record the Ca^2+^ flux, the SIET was demonstrated to be a sensitive and non-invasive approach for assaying MET channels [15].

The specific localization and function of the AE in hair cells are still controversial. With a polyclonal antibody against erythrocyte AE1, an early study in gerbils showed that AE1 was expressed in lateral walls of outer hair cells [16]. Nevertheless, studies in guinea pigs showed that AE2 but not AE1 was expressed in stereocilia and lateral walls of outer hair cells [17], [18]. A recent study in zebrafish revealed that aminoglycoside antibiotics and FM 1–43 uptake by neuromast hair cells was reduced in a *zslc4a1b* (zAE1b) mutant, suggesting that zAE1b is essential for the function of MET channels [19]. However, localization of zAE1b in hair cells has not been provided to link its function with MET channels. In the present study, *in situ* hybridization and immunocytochemistry were used to demonstrate the expression of zAE1b in stereocilia of hair cells where MET channels are located. The SIET was applied to demonstrate that MET channel-mediated Ca^2+^ influx can be suppressed by inhibiting AE1b function, which suggested that zAE1b in stereocilia is essential for the proper functioning of MET channels.

## Material and Methods

### Zebrafish

Adult zebrafish (*Danio rerio*, AB strain) were reared in circulating tap water at 28°C with a photoperiod of 14 h of light/10 h of dark at the Department of Anatomy and Cell Biology, Taipei Medical University, Taipei, Taiwan. The AB wild type zebrafish were provided by the Taiwan Zebrafish Core Facility at Academia Sinica (ZCAS). Fertilized eggs were incubated in artificial normal water (NW). The NW contained (in mM) 0.5 NaCl, 0.2 CaSO_4_, 0.2 MgSO_4_, 0.16 KH_2_PO_4_, and 0.16 K_2_HPO_4_ (pH 7.0). All of the incubating solutions were prepared by adding various salts (Sigma-Aldrich, St. Louis, MO, USA) to double-distilled water. During the experiments, larvae were not fed, and the NW was changed daily to ensure optimal water quality. The experimental protocols were approved by the Taipei Medical University Animal Care and Utilization Committee (approval no.: LAC-101–0189).

### Whole-mount *in situ* hybridization

For *in situ* hybridization, primers were designed following a previous study [20]. Fragments of *zslc4a1b* (nucleotides 110~812; NM_001168266) were obtained by a polymerase chain reaction (PCR) and inserted into the pGEM-T easy vector (Promega, Madison, WI, USA). The inserted fragments were amplified with the T7 and SP6 primers by a PCR, and the respective products were used as templates for *in vitro* transcription with T7 or SP6 RNA polymerase (Roche, Mannheim, Germany) in the presence of digoxigenin (DIG)-UTP (Roche, Mannheim, Germany), to respectively synthesize sense and antisense probes. DIG-labeled RNA probes were examined using RNA gels, and their quality and concentrations were determined using dot blot assays. Zebrafish larvae were anesthetized on ice and fixed with 4% paraformaldehyde in a phosphate-buffered saline (PBS; 1.4 mM NaCl, 0.2 mM KCl, 0.1 mM Na_2_HPO_4_, and 0.002 mM KH_2_PO_4_; pH 7.4) solution at 4°C overnight. Afterward, samples were washed with diethylpyrocarbonate (DEPC)-PBST (PBS with 0.1% Tween-20) several times (10 min/wash). Samples were subsequently incubated with hybridization buffer (HyB, 50% formamide, 5x SSC, and 0.1% Tween 20) at 65°C for 5 min, and then with HyB containing 500 μg/ml of yeast (t)RNA at 65°C for 4 h. Following overnight hybridization with 100 ng/ml of a DIG-labeled antisense or sense RNA probe, embryos were serially washed with 50% formamide-2x SSC (65°C for 20 min), 2x SSC (65°C for 10 min), 2x SSC (65°C for 10 min), 0.2x SSC (65°C for 30 min, twice), and PBST (room temperature for 10 min). Larvae were then immunoreacted with an alkaline phosphatase-coupled anti-DIG antibody (1:8000), and stained with nitro blue tetrazolium (NBT) (Roche, Mannheim, Germany) and 5-bromo-4-chloro-3-indolyl phosphate (NCIP) (Roche, Mannheim, Germany).

### Whole-mount immunocytochemistry

For immunocytochemistry, zebrafish larvae were fixed with 4% paraformaldehyde in a PBS solution at 4°C for 2 h. After being washed with PBS, samples were incubated with 3% bovine serum albumin (BSA) for 1 h to block nonspecific binding. Samples were then incubated overnight at 4°C with an anti-zebrafish AE1b polyclonal antibody [20] or an anti-actin monoclonal antibody (1: 100; Chemicon, Temecula, CA, USA). After washing with PBS for 20 min, samples were further incubated in Alexa Fluor 568 goat anti-rabbit (Molecular Probes; diluted 1: 200 with PBS) or goat anti-mouse antibodies (Molecular Probes; diluted 1:200 with PBS) for 2 h at room temperature. For double immunocytochemistry, samples were incubated overnight at 4°C with zAE1b polyclonal and parvalbumin monoclonal (1: 100; Millipore, Billerica, MA) antibodies. After washing, samples were further incubated in Alexa Fluor 568 goat anti rabbit and Alexa Fluor 488 goat anti-mouse for 2 h at room temperature. Images were obtained with an upright microscope (Imager M1, Carl Zeiss, Oberkochen, Germany) or a Leica TCS-SP5 confocal laser scanning microscope (Leica Lasertechnik, Heidelberg, Germany).

### Microinjection of antisense morpholino oligonucleotides (MOs)

The morpholino-modified antisense oligonucleotides were purchased from Gene Tools (Philomath, OR). MOs against zebrafish slc4a1b, which began at -1 bp and spanned the ATG ending at the 25th nucleotide position, 5’-CTTCGCAGACTCATTCATCTCCATG-3’, were prepared with sterile water. Standard control oligo provided by Gene tools was used as the control which has no target and no significant biological activity. The MO solution (1 ng per embryo) containing 0.1% phenol red (as a visualizing indicator) was injected into zebrafish embryos at the one- to two-cell stage using an IM-300 microinjection system (Narishigi Scientific Instrument Laboratory, Tokyo, Japan).

### Ion-selective microelectrodes

To construct ion-selective microelectrodes, glass capillary tubes (no. TW 150–4; World Precision Instruments, Sarasota, FL, USA) were pulled on a Sutter P-97 Flaming Brown pipette puller (Sutter Instruments, San Rafael, CA, USA) into micropipettes with tip diameters of 3~4 μm. The micropipettes were then baked at 120°C overnight and coated with dimethyl chlorosilane (Sigma-Aldrich) for 30 min. The micropipettes were backfilled with a 1-cm column of 100 mM CaCl_2_ for the Fluka Ca^2+^-selective microelectrode. The microelectrode was then frontloaded with a 20~30-μm column of Ca^2+^ ionophore I cocktail A (Sigma-Aldrich) to create a Ca^2+^-selective microelectrode.

### SIET

Details of the system were described in a previous report [15]. Briefly, the ion-selective microelectrode was connected to the main amplifier via an Ag/AgCl wire electrode holder and preamplifier (Applicable Electronics, East Falmouth, MA, USA), and the circuit was completed with a salt bridge (3 M KCl in 3% agarose connected to an Ag/AgCl wire). Oscillation and positioning of the microelectrode were performed with a step-wise motor-driven three-dimensional (3D) positioner (Applicable Electronics) which was attached to an Olympus upright microscope (BX-50WI, Olympus, Tokyo, Japan). The microscope was equipped with a digital camera (EOS 50D, Canon, Tokyo, Japan) which allowed images to be visualized on a monitor and recorded. Data acquisition, preliminary processing, and control of the 3D electrode positioner were performed with ASET software (Science Wares, East Falmouth, MA, USA).

### Calibration of ion-selective microelectrodes

To calibrate the ion-selective microelectrodes, the Nernstian property of each microelectrode was measured by placing the microelectrode in a series of standard solutions (Ca^2+^ microelectrode: 0.1, 1, 10, and 100 mM CaCl_2_). By plotting the voltage output of the probe against log [Ca^2+^] values, a linear regression yielded a Nernstian slope of 29 ± 0.45 (*n* = 10) for Ca^2+^. According to technical documents provided by Sigma, the selectivity coefficients of the Fluka Ca^2+^ ionophore I cocktail A is ~1000-times more selective to Ca^2+^ than to Mg^2+^.

### Measurement of Ca^2+^flux at neuromasts

The SIET was performed at room temperature (26~28°C) in a small plastic recording chamber filled with 1 ml of recording medium that contained NW, 300 μM MOPS buffer, and 0.1 mg/l ethyl 3-aminobenzoate methanesulfonate (tricaine, Sigma-Aldrich). The pH of the recording medium was adjusted to 7.0 by adding a NaOH or HCl solution. Before being measured, an anesthetized larva was positioned in the center of the chamber with its lateral side contacting the base of the chamber, and it was observed through a 60× water-immersion lens. Then the microelectrode was moved into the recording medium and positioned at the hair bundle of a neuromast to record Ca^2+^ activity. The microelectrode oscillated orthogonally for 10 m to deflect kinocilia and record Ca^2+^ influx. 5~10 replicate recordings were usually performed on a neuromast, and the median value was used to calculate ionic fluxes with ASET software (Applicable Electronics). Briefly, voltage gradients obtained from the ASET software were converted into concentration (activity) gradients using the following equation: ΔC = C_b_ × 10^(Δ*V/S*)^–C_b_; where ΔC is the concentration gradient between the two points measured in μmole·L^-1^·cm^-3^, C_b_ is the background ion concentration, calculated as the average of the concentration at each point measured in μmole·L^-1^, ΔV is the voltage gradient obtained from ASET in μV, and S is the Nernst slope of the electrode. The concentration gradient was subsequently converted into an (extracellular) ion flux using Fick’s law of diffusion in the following equation: J = D(ΔC) / ΔX; where J is the net flux of the ion in pmol·cm^-2^·s^-1^, D is the diffusion coefficient (of 8 × 10^–6^ cm^2^·s^-1^ for Ca^2+^), ΔC is the concentration gradient in pmol·cm^-3^, and ΔX is the distance between the two points measured in centimeters. The SIET was performed on first neuromast of posterior lateral line in zebrafish larvae.

### Drug preparation and treatment

4,4’-Diisothiocyano-2,2’-stilbenedisulfonic acid (DIDS; Sigma-Aldrich) was dissolved in DMSO to a stock concentration of 200 mM. Adequate stock was dissolved in NW to final concentrations of 200~500 μM. To prepare high Ca^2+^ NW, CaSO_4_ was dissolved in NW to 2 mM. Media were adjusted to pH 7.0. During SIET measurements, larvae were preincubated in drug medium for 30 min. Thereafter embryos were measured in recording medium without drugs.

### Statistical analysis

Data are expressed as the mean ± standard error (SE). Values from each condition were analyzed using a one-way analysis of variance (ANOVA) followed by Tukey’s pairwise comparisons. In all cases, significance was accepted at a level of 0.05. Student’s unpaired *t*-test (two-tailed) was used for simple comparisons of two means. Significance was set at level of 0.05.

## Results

### Localization of AE1b in neuromast hair cells of zebrafish larvae

**Fig 1:**
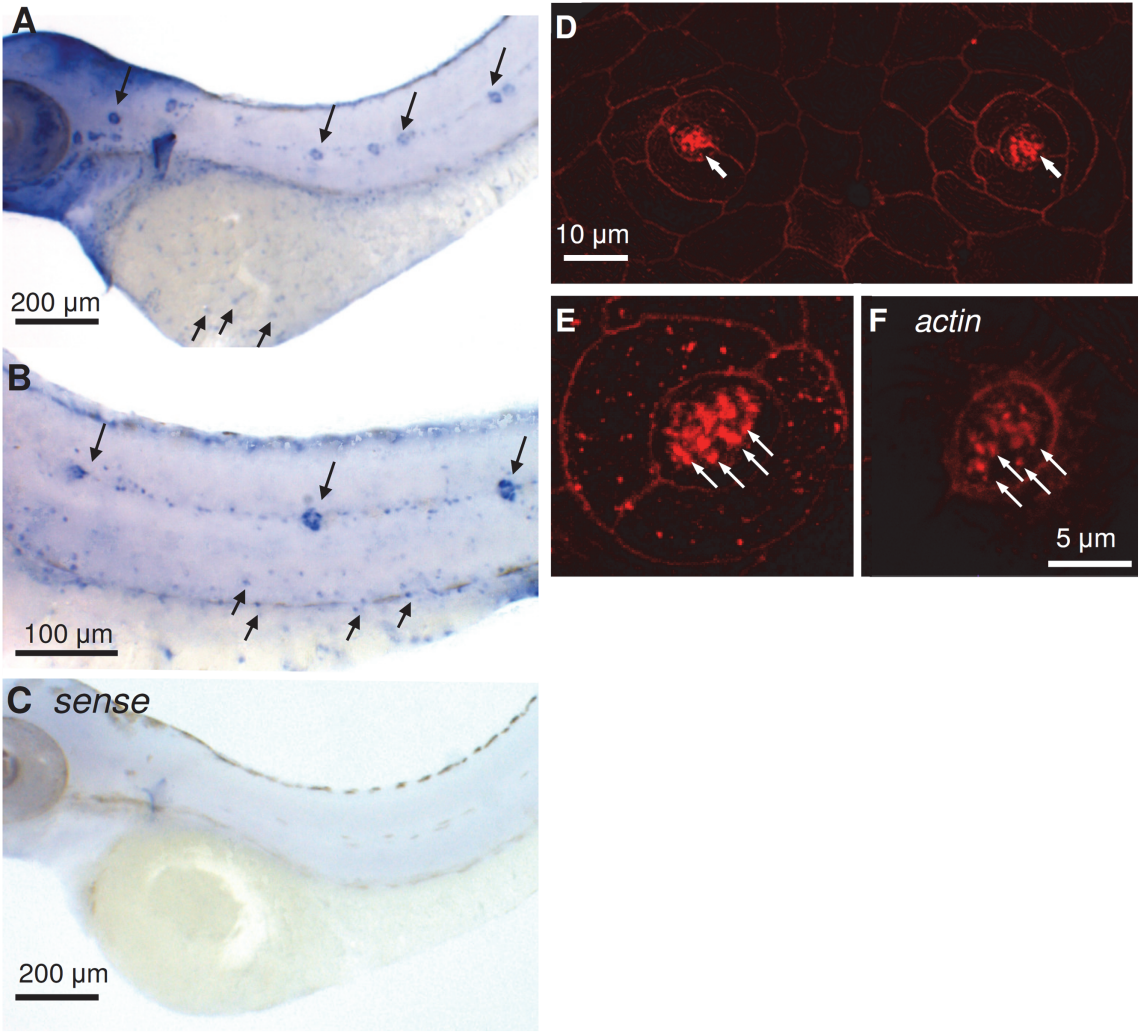
*In situ* hybridization and immunocytochemistry of anion exchanger 1b (AE1b) in zebrafish larvae. AE1b mRNA was expressed by ionocytes (short arrows) and neuromasts (long arrows) of 72-h post-fertilization (hpf) larvae (A, B). No signal was found in larval skin with the sense probe (C). Confocal images of lateral-line neuromasts with the zebrafish AE1b antibody (D, E). AE1b was expressed on the apical portion of neuromasts in 96-hpf larvae (arrows, D). Magnification of the neuromast apical membrane showing that AE1b was expressed in stereocilia (arrows, E). The morphology of stereocilia was verified by an actin antibody (arrows, F).

*In situ* hybridization was used to label *zslc4a1b* mRNA in 3-day post-fertilization (3-dpf) zebrafish larvae, and results showed that *zslc4a1b* was expressed in neuromasts distributed in discrete lines over the body surface (long arrows in Fig. 1A, B) and ionocytes scattered on the skin and yolk sac (short arrows in Fig. 1A, B; [20]). Signals were not found in the negative control with sense probes (Fig. 1C). In addition, zAE1b immunohistochemistry with a zebrafish-specific antibody was used to localize zAE1b protein expression in larvae, and confocal images revealed fluorescent signals in the stereocilia of neuromast hair cells (Fig. 1D, E). The stereocilia of hair cells was marked by actin immunohistochemistry (Fig. 1F).

### Effect of zAE1b knockdown on neuromast hair cell

**Fig 2:**
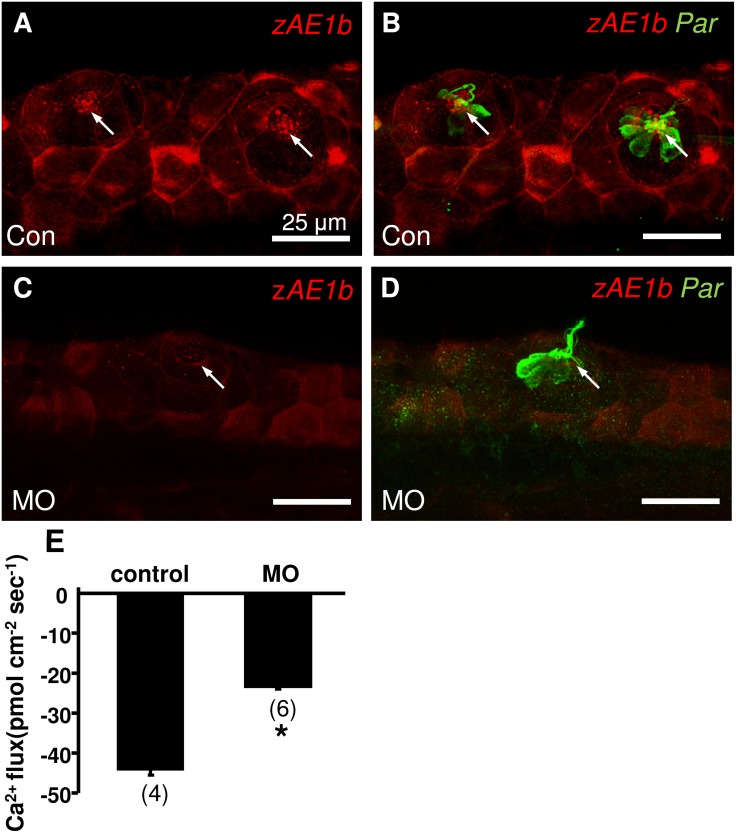
Effects of morpholino knockdown of zAE1b on neuromast hair cell. Double staining of zAE1b (red; A-C) and parvalbumin (Par; green; B, D) antibodies was conducted in control (Con; A, B) and morpholino knockdown (MO; C, D) 3 dpf larvae. B and D: merged images of zAE1b and Par protein signals. (E) The Ca^2+^ influx of neuromast hair cells in zAE1b morpholino knockdown larvae was significantly decreased. Data are presented as the mean ± SE. *Significant difference (Student’s *t* test, p<0.05). Numbers in parentheses are numbers of neuromasts.

Specific MOs were used to knockdown the protein expression of zAE1b to examine the specificity of zAE1b antibody and hair cell function. Following the previous study [20], 1 ng/embryo of zAE1b and control MO were injected into fertilized eggs. The phenotype of MO injected larvae (morphants) showed no difference with that of wildtype larvae (data not show). Parvalbumin antibody was used to label neuromast hair cells in larvae, and no significant difference was found in the morphology and number of hair cells between control and morphants (Fig. 2B, D). However, the signal of zAE1b in stereocilia was remarkably reduced in zAE1b morphants (Fig. 2A, C). The MET channel-mediated Ca^2+^ influx at neuromast of control and morphants was measured with the SIET. The Ca^2+^ influx (negative values represent influx of Ca^2+^) was significantly decreased in zAE1b morphants (Fig. 2E).

### Effects of DIDS on hair cell Ca^2+^ influx

**Fig 3:**
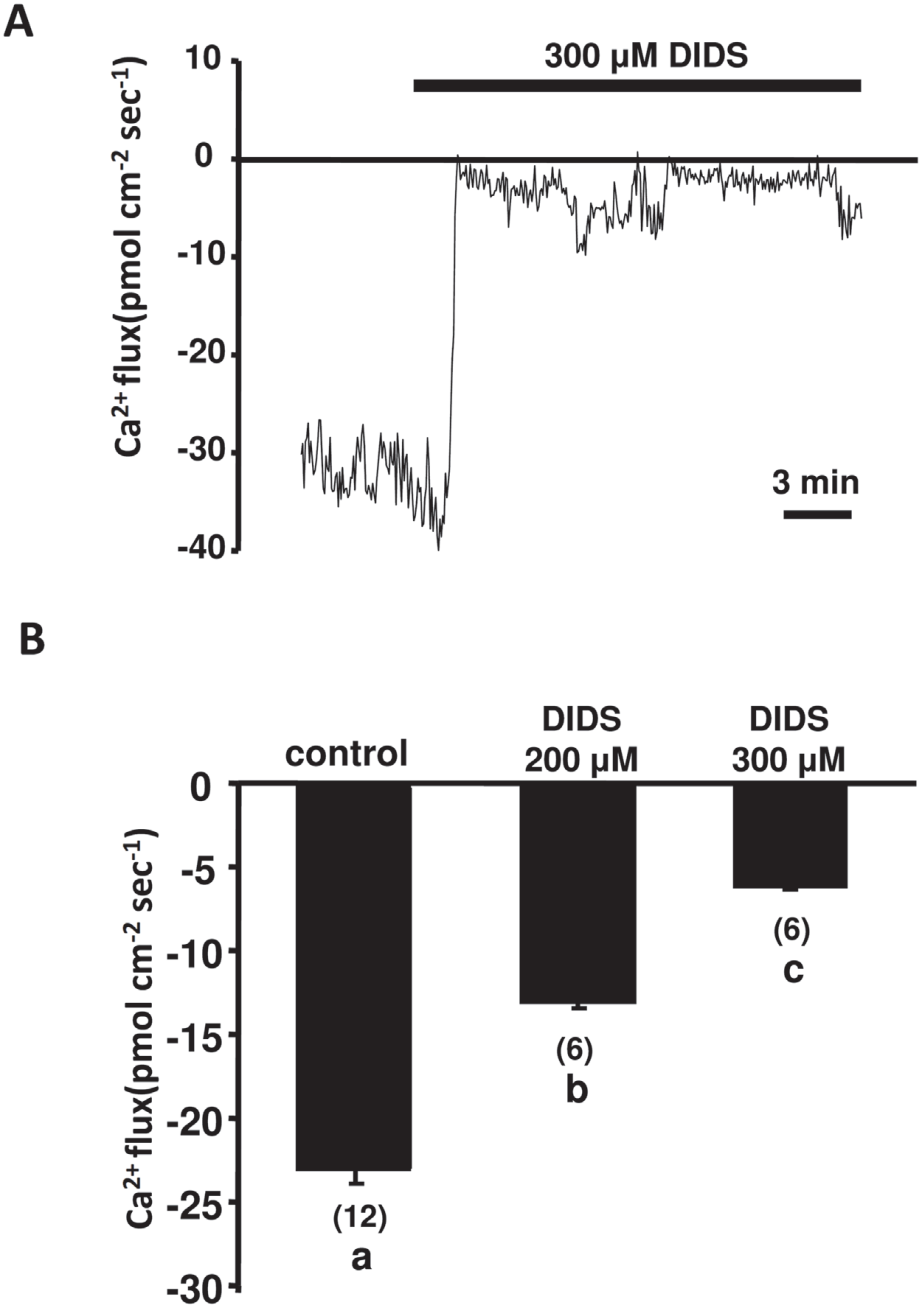
Effect of DIDS on Ca^2+^ influx of neuromasts. (A) Sequential recording of Ca^2+^ influx of neuromast hair cells in 96-hpf larvae before and after the addition of 300 μM DIDS. (B) Larvae were pre-incubated in normal water with 200 or 300 μM DIDS for 30 min. Then the influx was recorded in normal recording medium without DIDS. Data are presented as the mean ± SE. ^a, b, c^ Indicate a significant difference (by one-way ANOVA, Tukey’s comparison, *p* < 0.05). Numbers in parentheses are numbers of neuromasts.

Real-time recordings of SIET showed the Ca^2+^ influx at neuromasts before and after addition of 300 μM DIDS. The Ca^2+^ influx was dramatically suppressed after the addition of DIDS (Fig. 3A). Dose-dependent inhibition of Ca^2+^ influx was found in larvae pretreated with 200 and 300 μM DIDS for 30 min (Fig. 3B).

### Addition of Ca^2+^ neutralized the effect of DIDS

**Fig 4:**
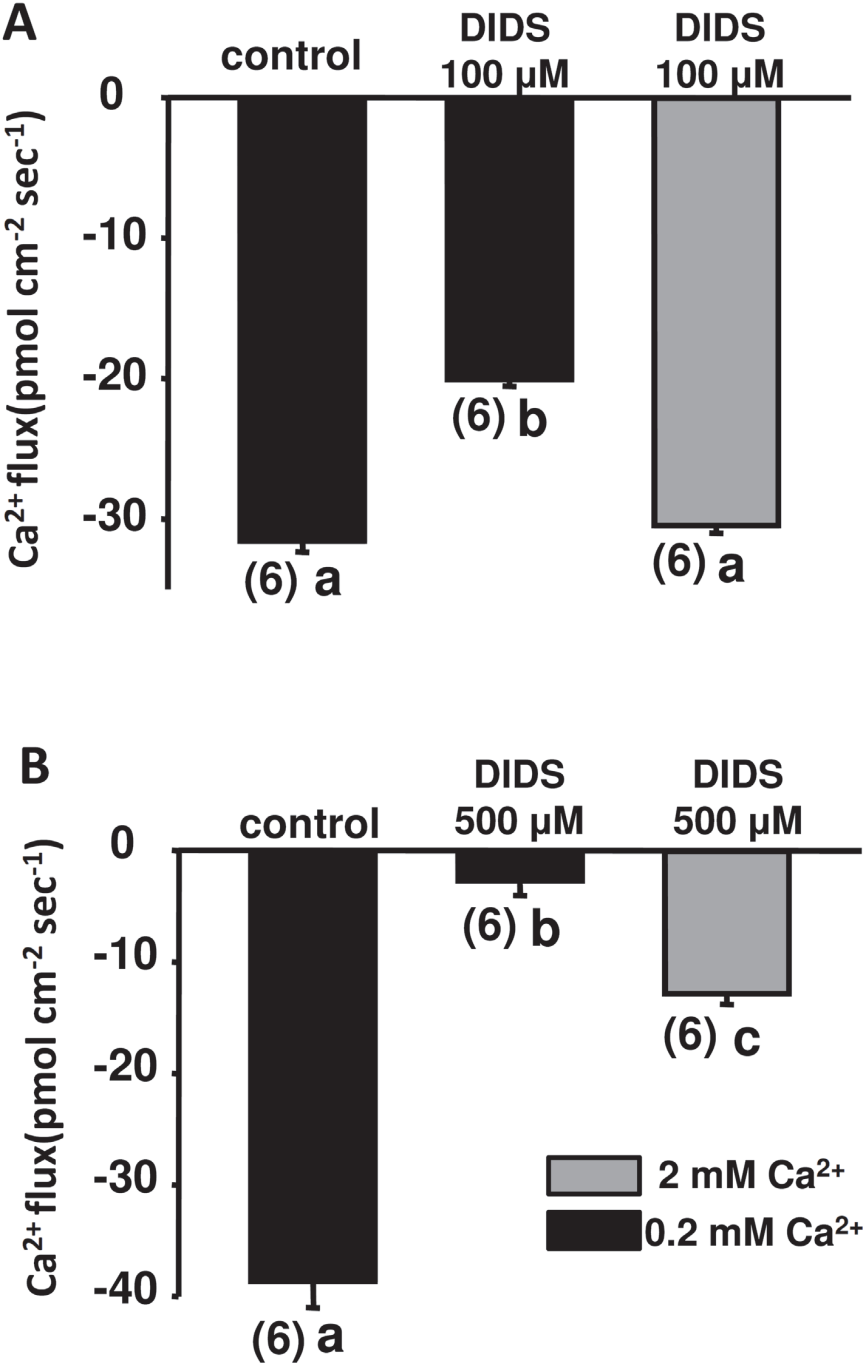
Effects of external Ca^2+^ on inhibition by DIDS. Embryos were pre-incubated in normal water (black bar; 0.2 mM Ca^2+^) or high-Ca^2+^ normal water (gray bar; 2 mM Ca^2+^) with 100 (A) or 500 μM DIDS (B) for 30 min. Ca^2+^ influx was recorded in normal recording medium. Data are presented as the mean ± SE. ^a, b, c^ Indicate a significant difference (by one-way ANOVA, Tukey’s comparison, *p* < 0.05). Numbers in parentheses are numbers of neuromasts.

To test if raising the external Ca^2+^ level could neutralize the inhibition by DIDS, larvae were preincubated in DIDS medium with 0.2 or 2 mM Ca^2+^ for 30 min, and then the Ca^2+^ influx was measured in recording medium with 0.2 mM Ca^2+^. Results showed that 2 mM Ca^2+^ completely neutralized inhibition by 100 μM DIDS (Fig. 4A), and partially neutralized inhibition by 500 μM DIDS (Fig. 4B).

## Discussion

A recent study showed that loss of function of AE1b (*zslc4a1b*) in a zebrafish mutant (persephone) reduced hair cell death caused by exposure to aminoglycoside antibiotics; pharmacological blockage of the AE with DIDS and SITS also protected hair cells against aminoglycosides [19]. Both the persephone mutant and DIDS-treated wild-type larvae showed reduced uptake of aminoglycosides and FM1–43, suggesting a potential impact on mechanotransduction associated activity in the mutant [19]. However, localization of AE1b in zebrafish was not revealed in that study to support a potential functional link between AE1b and the MET channel.

In a previous study, a specific antibody against zebrafish AE1b was generated to localize zAE1b in the basolateral membrane of skin ionocytes (H^+^-ATPase-rich cells) which is responsible for ion and acid-base regulation [20], [21]. Specificity of the antibody was confirmed by Western blotting and morpholino knockdown experiments in that study [20]. In the present study, we used the same antibody to show that zAE1b is also expressed in the stereocilia of neuromast hair cells where are considered to be major bundles for mechanotransduction and location of MET channels (Fig. 1; [7], [8]). The signal of zAE1b in stereocilia was reduced in zAE1b morphants demonstrating the specificity of zAE1b antibody (Fig. 2). Most importantly, this localization evidence of AE1b in hair bundles supports a potential functional link between AE1b and the MET channel.

The SIET was designed to detect very weak ion fluxes near cells and tissues, such as H^+^, Cl^-^, Na^+^, and Ca^2+^ fluxes near retina cells [22], kidney cell lines [23], mouse blastocysts [24], and embryonic skin of zebrafish [21], [25], [26]. In our previous study, we applied the SIET for the first time to analyze MET channel-mediated Ca^2+^ influx at the hair bundle of zebrafish neuromasts [15]. Using a Ca^2+^-selective microelectrode to deflect hair bundles and simultaneously record the Ca^2+^ flux, the inward Ca^2+^ flux was detected at stereocilia of hair cells. As little as 10 µM aminoglycoside antibiotics (neomycin and gentamicin) was found to effectively block Ca^2+^ influx within 10 min, showing the SIET to be a sensitive approach for functionally assaying MET channels in intact zebrafish [15]. In this study, the MET channel mediated Ca^2+^ influx measured with the SIET was significantly decreased in zAE1b morphant (Fig. 2). Our result was in accordance with the previous study which demonstrated an impact on mechanotransduction in zAE1b mutant [19]. Furthermore, Ca^2+^ influx measured by the SIET was effectively blocked by DIDS, suggesting that AE1b in stereocilia is required for the proper functioning of MET channels. DIDS is widely used as an inhibitor of anion transporters and channels, such as Cl^-^ channels and Cl^-^/HCO_3_^-^ exchangers [27]. In the zebrafish lateral line, 200 μM DIDS significantly reduced the uptake of aminoglycosides by hair cells [19]. In our results, we showed that 100 μM DIDS significantly reduced the MET channel-mediated Ca^2+^ influx (Fig. 4A).

AE1 is a multifunctional protein which plays a key role in the CO_2_/HCO_3_^-^ buffering system of the blood and maintains acid-base homeostasis in the kidneys [28]. In addition to membrane transport, AE1 interacts with the cytoskeleton to give erythrocytes sufficient flexibility to pass through capillaries [1], [28]. AE1 also forms a “multiprotein complex” with other proteins such as rhesus polypeptides (RhD and RhCE), carbonate anhydrase (CA), protein 4.1 (which links AE1 to ankyrin and the cytoskeleton) [1], [28], [29]. Studies in hair cells of gerbils and guinea pigs suggested that AE1 (band 3) links to the cytoskeleton and provides structural stability for hair bundles [16], [17]. In addition, the connection between the cytoskeleton and MET channels was found to be critical for activation of MET channels [30]. Therefore, AE1b in hair bundles of zebrafish might form a “multiprotein complex” with MET channels, the cytoskeleton, and other proteins, and thus loss of functional AE1b impairs MET channel activity.

Alternatively, AE1b might play a critical role in maintaining hair bundle pH, and a proper pH is required for mechanotransduction by hair cells. In mammalian kidneys, both the SLC4 and SLC26 families are Cl^-^/HCO_3_^-^ exchangers and are critical for maintaining acid-base homeostasis [31]. *slc26a4* (pendrin) was found in tissues of the inner ear including apical membranes of strial spindle cells, the spiral prominence, and outer sulcus epithelial cells [32], [33]. It was proposed that pendrin secrets HCO_3_^-^ to maintain endolymph alkalinity, and deletion of pendrin leads to acidification of the endolymph and degeneration of hair cells [32], [33]. In addition, the plasma membrane calcium ATPase (PMCA) in stereocilia was suggested to pump Ca^2+^ out of stereocilia and simultaneously carry H^+^ into stereocilia [34], [35]. Loss-of-function of AE1b might alter intracellular pH balance, affect PMCA function and eventually suppress MET channel. The exact function of AE1b in hair bundles of zebrafish is still uncertain and needs to be further investigated.

Experiments with mouse cochlea and zebrafish neuromasts showed that aminoglycoside-provoked hair cell damage was suppressed by elevating the extracellular Ca^2+^ concentration [36], [37]. It was suggested that elevating external Ca^2+^ inactivates MET channels and thus decreases aminoglycoside entry [15], [38]. In the present study, we also found that high extracellular Ca^2+^ neutralized the inhibition of Ca^2+^ influx by DIDS, suggesting that AE1b and MET channels might interact with each other only when MET channels are activated. High external Ca^2+^ might inactivate MET channels and delink AE1b and MET channels.
